# Supplementary material for: Causal interplay between lactose intolerance and gut microbiota: a combined bidirectional Mendelian randomization and in vivo validation study
Source: Front Nutr. 2026 Jun 1;13:1803337. doi: 10.3389/fnut.2026.1803337 (PMC13265576; doi:10.3389/fnut.2026.1803337)
Supplement: Supplementary file 1 [file Data_Sheet_1.zip › supplementary materials/Forward/leave one out cross validation/ebi-a-GCST90027477.finngen_R12_E4_LACTONAS.pdf]

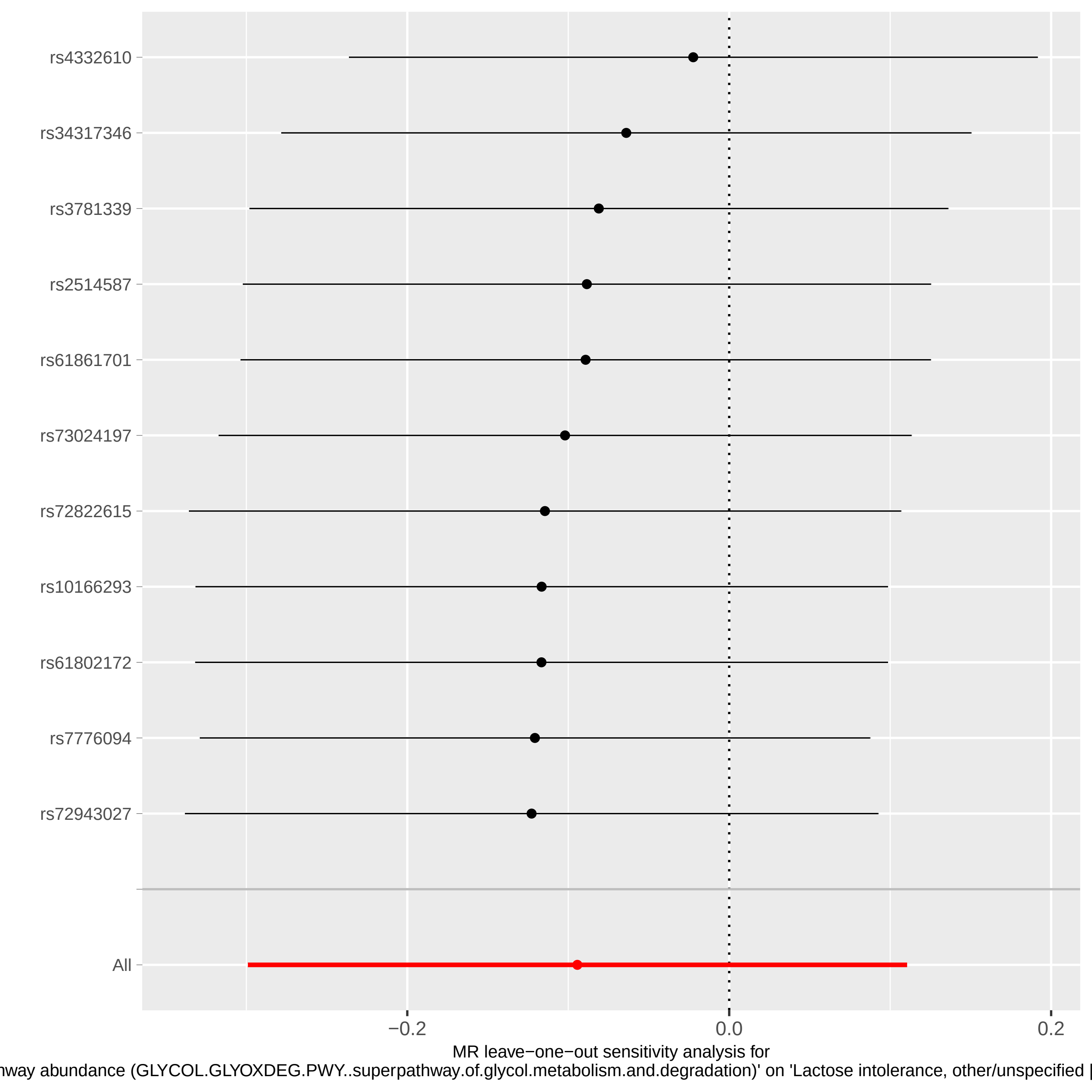

rs4332610
rs34317346
rs3781339
rs2514587
rs61861701
rs73024197
rs72822615
rs10166293
rs61802172
rs7776094
rs72943027
All
−0.2
0.0
0.2
MR leave−one−out sensitivity analysis for
hway abundance (GLYCOL.GLYOXDEG.PWY..superpathway.of.glycol.metabolism.and.degradation)' on 'Lactose intolerance, other/unspecified
